# Development and Validation of a Prediction Model of the Risk of Pneumonia in Patients with SARS-CoV-2 Infection

**DOI:** 10.1155/2023/6696048

**Published:** 2023-07-18

**Authors:** Xi Yi, Daiyan Fu, Guiliang Wang, Lile Wang, Jirong Li

**Affiliations:** ^1^Department of Radiology, Hunan Provincial People's Hospital/The First Affiliated Hospital of Hunan Normal University, Changsha 410016, China; ^2^Department of Respiratory Medicine, Hunan Provincial People's Hospital/The First Affiliated Hospital of Hunan Normal University, Changsha 410016, China

## Abstract

**Objective:**

To establish a prediction model of pneumonia risk in SARS-CoV-2-infected patients to reduce unnecessary chest CT scans.

**Materials and Methods:**

The model was constructed based on a retrospective cohort study. We selected SARS-CoV-2 test-positive patients and collected their clinical data and chest CT images from the outpatient and emergency departments of Hunan Provincial People's Hospital, China. Univariate and multivariate logistic regression and least absolute shrinkage and selection operator (LASSO) regression were utilized to identify predictors of pneumonia risk for patients infected with SARS-CoV-2. These predictors were then incorporated into a nomogram to establish the model. To ensure its performance, the model was evaluated from the aspects of discrimination, calibration, and clinical validity. In addition, a smoothed curve was fitted using a generalized additive model (GAM) to explore the association between the pneumonia grade and the model's predicted probability of pneumonia.

**Results:**

We selected 299 SARS-CoV-2 test-positive patients, of whom 205 cases were in the training cohort and 94 cases were in the validation cohort. Age, CRP natural log-transformed value (InCRP), and monocyte percentage (%Mon) were found to be valid predictors of pneumonia risk. This predictive model achieved good discrimination of AUC in the training and validation cohorts which was 0.7820 (95% CI: 0.7254–0.8439) and 0.8432 (95% CI: 0.7588–0.9151), respectively. At the cut-off value of 0.5, it had a sensitivity and specificity of 70.75% and 66.33% in the training cohort and 76.09% and 73.91% in the validation cohort, respectively. With suitable calibration accuracy shown in calibration curves, decision curve analysis indicated high clinical value in predicting pneumonia probability in SARS-CoV-2-infected patients. The probability of pneumonia predicted by the model was positively correlated with the actual pneumonia classification.

**Conclusion:**

This study has developed a pneumonia risk prediction model that can be utilized for diagnostic purposes in predicting the probability of pneumonia in patients infected with SARS-CoV-2.

## 1. Introduction

SARS-CoV-2 infection has prevailed globally since 2020, accounting for recurring quarantines in many countries. It has significantly impacted public health and the global economy [1, 2]. As of 10 February 2023, there have been 755,385,709 confirmed cases of COVID-19 reported to WHO globally, including 6,833,388 deaths. Omicron, the mutant strain, entered the community in November 2021 and is far more contagious and escape-resistant than the previous variants of concern (VOC), like Delta [3–8]. At the beginning of 2022, the Omicron version quickly surpasses the Delta variant as the prevalent strain worldwide [9].

During the early period of the COVID-19 pandemic, SARS-CoV-2 primarily affected the lung and caused pneumonia [10–13]. As one of the most representative and accurate diagnostic methods for COVID-19 [14], chest computed tomography (CT) scans are widely used in mainland China.

However, recent studies have demonstrated that the most recent VOC, Omicron is much less likely to cause pulmonary infections [3–5, 15, 16], suggesting potential implications for adapting management strategies for these infections.

In clinical practice, we found that due to the apprehension of contracting severe pneumonia from the SARS-CoV-2, many people with mild symptoms are choosing to receive CT scans, causing excessive CT scans and putting a strain on the availability of healthcare resources, which is particularly true when SARS-CoV-2 localized epidemic outbreaks occur. Therefore, a strategy to evaluate the risk of pneumonia among recently infected people is essential to ensure the efficient use of medical resources and decrease unnecessary exposure to electromagnetic radiation.

This study is to improve the classification of pneumonia risk in individuals with the most recent VOC of SARS-CoV-2 infections. In this way, it can not only reduce the overuse of CT scans and nonessential ionizing radiation in individuals but also reduce the associated financial burden on patients and optimize the allocation of medical resources. As a result, we have developed and externally validated a pneumonia risk prediction model based on general patient data and blood routine tests, which meets the needs of the new phase of COVID-19 epidemic control.

## 2. Material and Methods

### 2.1. Materials

**Figure 1:**
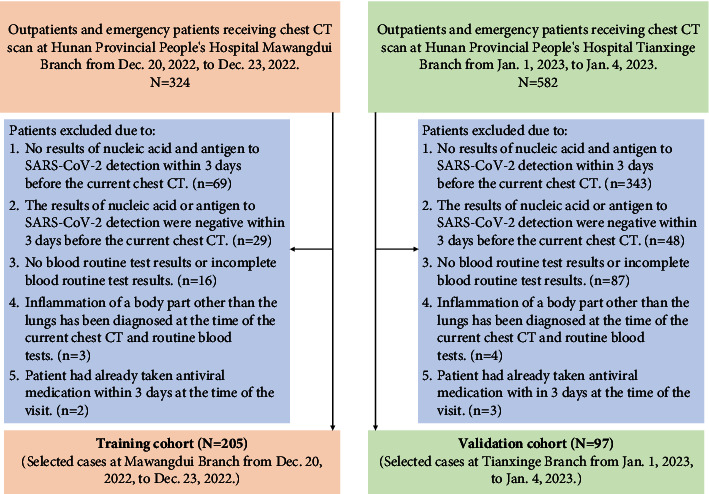
Instructions for enrolling in the training cohort and validation cohort cases.

A retrospective analysis was performed on the clinical data of SARS-CoV-2 test-positive patients who visited outpatient and emergency departments and underwent chest CT scans at the Mawangdui Branch of Hunan Provincial People's Hospital from 20 December 2022 to 23 December 2022 and at the Tianxinge Branch of Hunan Provincial People's Hospital from 1 January 2023 to 4 January 2023. The inclusion criteria were as follows: (a) attendance as an outpatient or emergency (not including inpatients); (b) patients had completed chest CT scans, and CT imaging data were available; (c) SARS-CoV-2 infection positive was diagnosed by antigen test or nucleic acid test within 3 days before the current chest CT; (d) complete blood routine examination results were obtained. The exclusion criteria were as follows: (a) inflammation of a body part other than the lungs had been diagnosed at the time of the current blood routine tests; (2) the patient was already on antiviral medication at the time of the visit. The patient recruitment pathway is detailed in Figure 1.

The study was conducted in accordance with the Declaration of Helsinki. It was approved by the Medical Ethics Committee of Hunan Provincial People's Hospital (The First Affiliated Hospital of Hunan Normal University), and patient informed consent was waived for this retrospective analysis.

### 2.2. Methods

#### 2.2.1. Device Parameters and Image Analysis

At the Mawangdui Branch (training cohort) of Hunan Provincial People's Hospital, CT scans were performed with a United Imaging uCT 760GE 128-slice CT using the following parameters: field of view (FOV), 230 mm × 230 mm; layer thickness, 5 mm; and layer spacing, 5 mm. At the Tianxinge Branch (validation cohort) of Hunan Provincial People's Hospital, CT scans were performed with a United Imaging uCT 860 160-slice CT or a United Imaging uCT 960 + 640-slice CT using the following parameters: field of view (FOV), 230 mm × 230 mm; layer thickness, 5 mm; and layer spacing, 5 mm. Two attending radiologists conducted image analysis separately, and the final decision in case of a dispute was determined by consultation between the two physicians. CT diagnosis of COVID-19 was referred to the report published by the RSNA [17]. Typical findings were as follows: peripheral distribution, ground-glass opacity, fine reticular opacity, vascular thickening, and reverse halo sign. Patients with pneumonia were also classified into grades 0, 1, 2, 3, and 4 according to the extent and distribution of lung involvement (no lung involvement was categorized as grade 0).

#### 2.2.2. Statistical Analysis and Construction and Evaluation of Predictive Models

Statistical analysis was performed using Empower Stats, version 5.0 (https://www.empowerstats.com, X&Y Solutions, Inc., Boston, MA, USA), R statistical software, version 4.2.0 (https://www.R-project.org, The R Foundation), and the SPSS statistical software, version 27.0 (SPSS Inc., Chicago, IL, USA) with continuity variables expressed as medians (min, max) and categorical variables expressed as frequencies (percentages). Kruskal–Wallis rank sum test or Fisher's exact probability test was used to compare differences between groups of continuity variables. The Chi-square test was used for comparisons of categorical variables. After the natural log transformation of some continuity variables, to reduce irrelevant and redundant information, the predictor variables of the training cohort were filtered using both “univariate and then multivariate logistic regression” and “least absolute shrinkage and selection operator (LASSO)” methods. The variables selected by both screening methods were used as the final predictor variables. The prediction model was constructed based on multivariate logistic regression and was presented in a nomogram. The ROC curves were used, and 500 in eternal resamples were performed by Bootstrap to evaluate the discrimination of the pneumonia risk model between the training and validation cohorts. DeLong test and integrated discrimination improvement index (IDI) were used to compare the AUC of the pneumonia risk model with the AUCs for predictors incorporated in the model alone. Calibration curves were plotted to assess the calibration of the model. The clinical validity of the model was evaluated by the net benefit of DCA at different threshold probabilities. In addition, a smoothed curve was fitted using a generalized additive model (GAM) to explore the relationship between the pneumonia grade and the model's predicted probability of pneumonia. A difference of *P* < 0.05 was considered statistically significant.

## 3. Results

### 3.1. General Information

A total of 205 patients were enrolled in the training cohort, of which 105 cases (51.22%) were female and 100 cases (48.78%) were male, 99 cases (48.29%) without pneumonia and 106 cases (51.71%) with pneumonia. The median age of the training cohort was 47 years old, the youngest being 14 and the oldest being 97; a total of 94 cases were enrolled in the validation cohort, of which 60 (63.83%) were female and 34 (36.17%) were male, 47 (50.00%) were without pneumonia, and 47 (50.00%) were with pneumonia. The median age of the validation cohort was 56 years old, the youngest being 2 and the oldest 89; the distribution of the remaining baseline indicators is shown in Table 1.

### 3.2. Predictor Variable Screening Results

**Figure 2:**
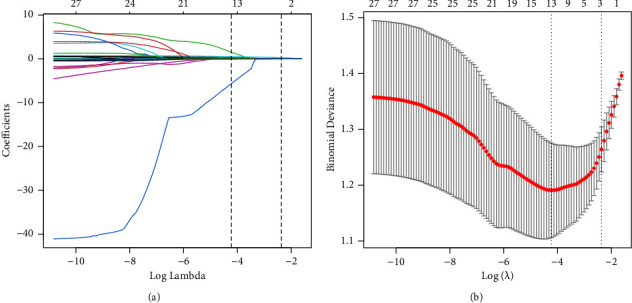
(a) LASSO regression coefficient path diagram; (b) LASSO regression cross-validation curve. Three predictors with nonzero coefficients were selected by LASSO regression (screening lambda by 10-fold cross-validation, based on lambda. 1se, i.e., the maximum lambda corresponding to an error mean within one standard deviation of the minimum): age, InCRP, %Mon.

Among the baseline indicators in the training cohort, univariate logistic regression identified the following factors as possible predictors (*P* < 0.1): age, white blood cells (WBC), red blood cells (RBC), neutrophils percentage (%Neu), neutrophils number (#Neu), lymphocytes percentage (%Lymph), monocytes percentage (%Mon), red cell distribution width-standard deviation (RDW-SD), platelet distribution width (PDW), mean platelet volume (MPV), platelet large cell ratio (P-LCR), CRP natural log-transformed value (InCRP), eosinophils percentage (%Eos), basophils percentage (%Bas), basophils number (#Bas). Further multivariate logistic regression showed age, CRP natural log-transformed value (InCRP), neutrophils percentage (%Neu), and monocytes percentage (%Mon) as independent predictors (*P* < 0.05) (Table 2). Lasso regression selected three predictors with nonzero coefficients: age, InCRP, %Mon (Figure 2) (screening lambda by 10-fold cross-validation, based on lambda. 1se, i.e., the maximum lambda corresponding to an error mean within one standard deviation of the minimum). To lessen irrelevant and redundant information, the variables age, InCRP, and %Mon selected by both screening methods were taken as the final predictor variables.

### 3.3. Construction and Evaluation of the Nomogram Prediction Model

**Figure 3:**
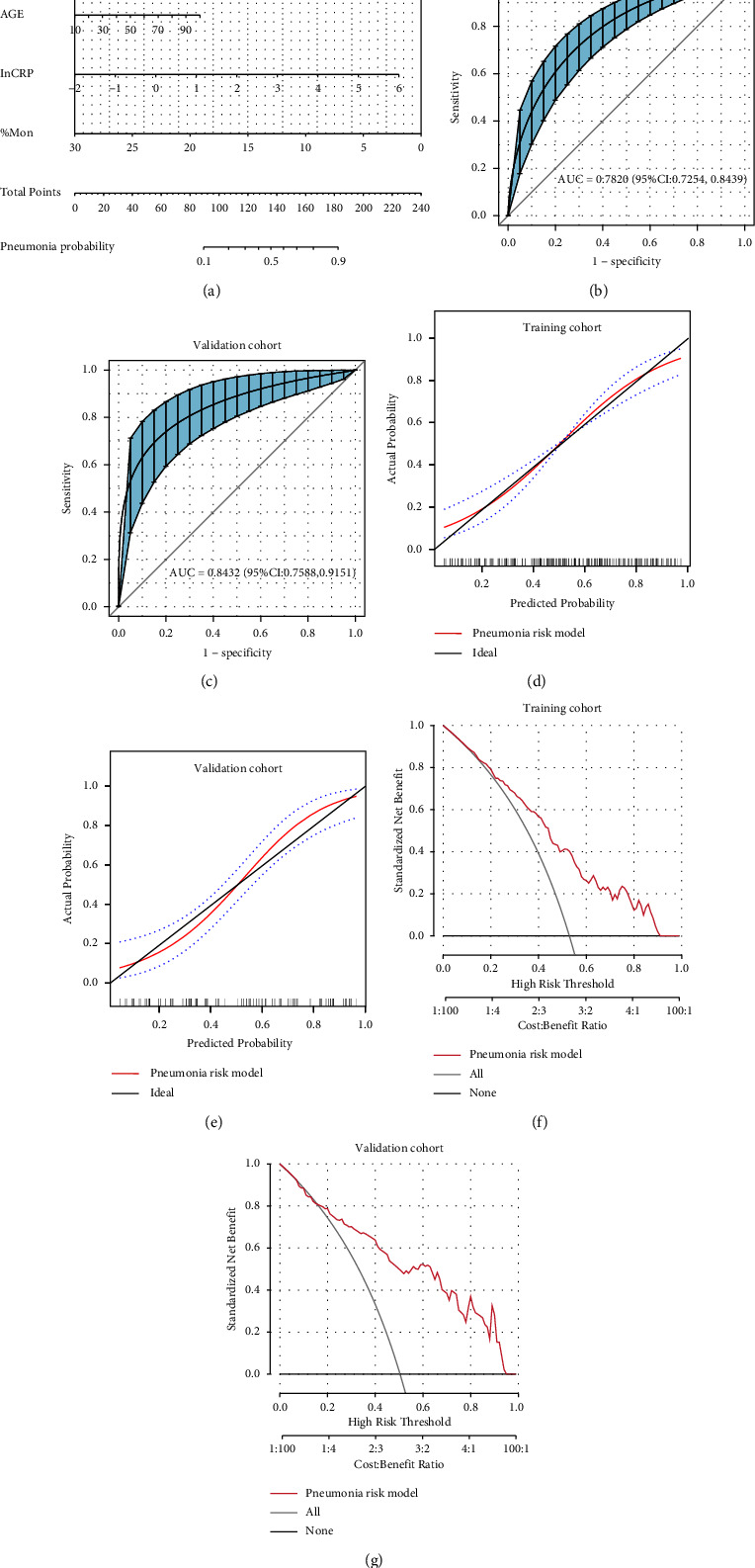
(a) Nomogram of the pneumonia risk model; (b–g): ROC curves (bootstrap = 500 times), calibration curves, and DCA curves of the pneumonia risk model in the training and validation cohorts. The ROC curves show good discrimination of the pneumonia risk model in both the training and validation cohorts. The calibration curves showed that the pneumonia risk model has good calibration accuracy. The decision curve analysis showed that the pneumonia risk model has high clinical value in predicting the probability of pneumonia in SARS-CoV-2-infected patients.

**Figure 4:**
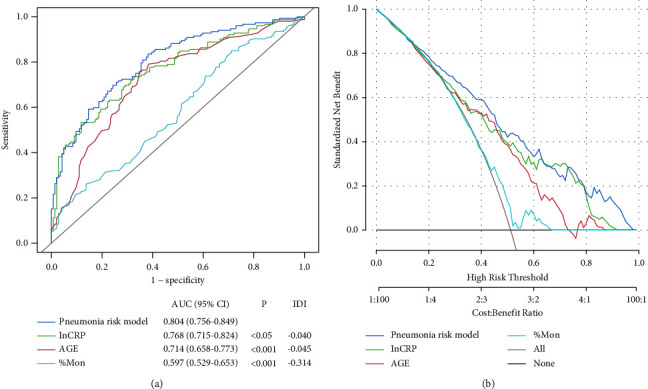
Comparison of the models in the whole study cohort. (a) Receiver operator characteristic curves of the models are presented to compare their discriminatory accuracy for predicting pneumonia risk. *P* values show the AUC for the pneumonia risk model versus the AUCs for predictors incorporated in the model alone; the predictive ability of the predictors in the model individually and the overall predictive power of the pneumonia risk model are contrasted via IDI. (b) Decision curve analyses comparing the net benefit of the nomogram of the pneumonia risk model versus the other variables incorporated in the nomogram alone are shown. AUC: area under the curve; CI: confidence interval; IDI: integrated discrimination improvement.

Multivariable logistic regression analysis established a nomogram model based on the final selected predictor variables (Figure 3(a)). The AUC of the pneumonia risk model was 0.7820 (95% CI: 0.7254–0.8439) in the training cohort and 0.8432 (95% CI: 0.7588–0.9151) in the validation cohort (Figures 3(b) and 3(c)); at the cut-off value of 0.5, the sensitivity and specificity of the pneumonia risk model were 70.75%, 66.33% (training cohort), 76.09%, and 73.91% (validation cohort), respectively; the calibration curve showed good agreement between the predicted probability of pneumonia from the pneumonia risk model and the actually observed probability. Decision curve analysis (DCA) showed good clinical validity of the pneumonia risk model in the training and validation cohort (Figures 3(f) and 3(g)). Other diagnostic parameters of the model are shown in Table 3. A comparison of the AUC and DCA for the pneumonia risk model, with predictors incorporated in the model alone in the whole study cohort, is illustrated in Figure 4, which shows that the pneumonia risk model combining multiple predictors has better diagnostic performance than a single predictor.

### 3.4. Correlation between the Predicted Probability of Pneumonia Risk and Pneumonia Grade

**Figure 5:**
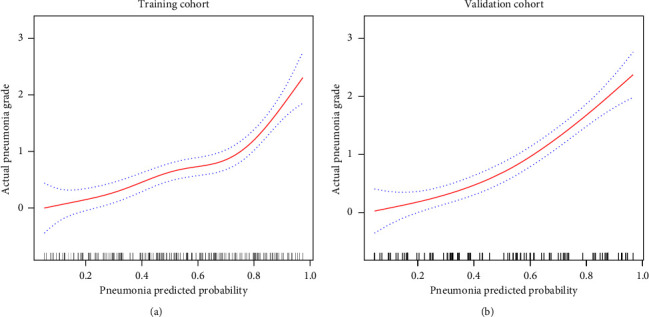
Correlation between the predicted probability of pneumonia risk and pneumonia grade ((a) training cohort; (b) validation cohort). A positive linear correlation was found between the predicted pneumonia probability of the pneumonia risk model and pneumonia grade using GAM.

**Figure 6:**
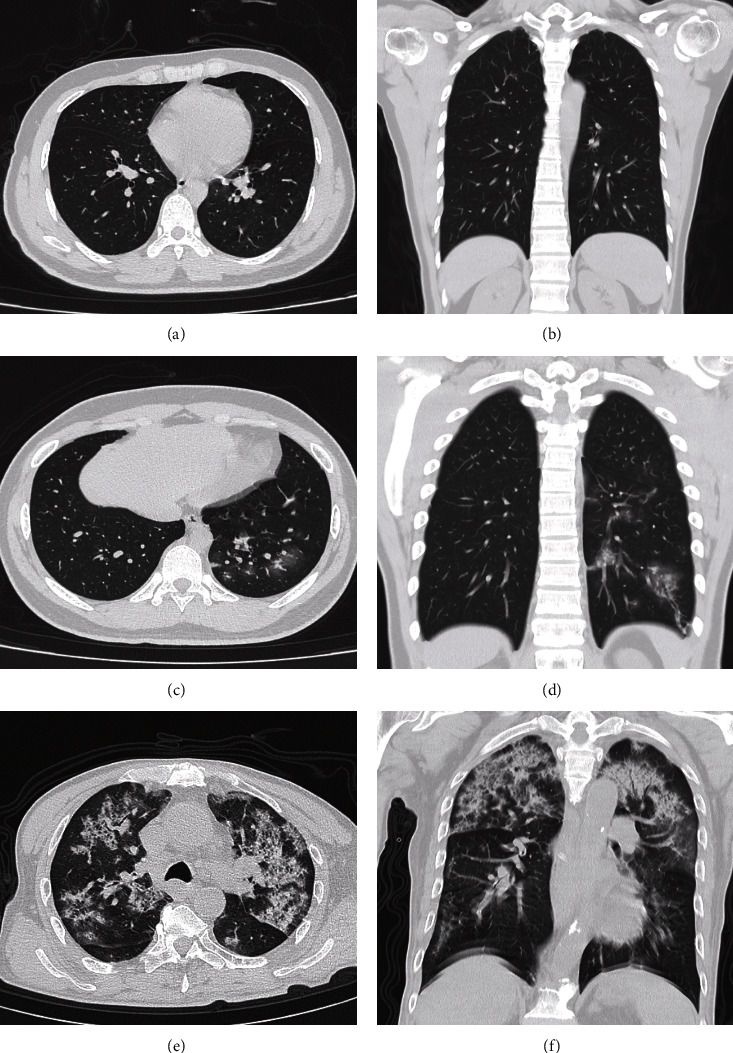
Examples of the pneumonia risk model applications. (a and b): A 32-year-old male presented with a 1-day history of fever with a maximum temperature of 39.2°C. At the time of presentation, he was confirmed positive by nucleic acid testing for SARS-CoV-2. His routine blood test showed a CPR of 14.08 (InCRP = 2.70) and %Mon of 26.50. Considering his age of 32, the patient got a total of 75 points according to our pneumonia risk prediction model, with a pneumonia risk prediction probability of <0.1. The patient underwent a CT chest scan, which showed no abnormal findings. (c and d): Male, 17 years old, presented 4 days ago with a fever with a maximum temperature of 39.0°C. On presentation, he was confirmed positive by nucleic acid testing for SRRS-CoV-2. His routine blood test showed a CPR of 82.45 (InCRP = 4.41) with a %Mon of 8.30. Considering his age of 17, the patient had total points of 152 according to our pneumonia risk prediction model, with a pneumonia risk prediction probability of 0.68. The patient underwent a chest CT, which showed multiple lamellar ground-glass opacities in the lower lobe of the left lung, with a peripheral distribution and thickened blood vessels within the lesion. (e and f): A 63-year-old male with a 1-week history of malaise was confirmed to be nucleic acid test positive for SARS-CoV-2 on presentation. His routine blood test showed a CRP of 259.68 (InCRP = 5.56) with a %Mon of 5.00. Considering his age of 63, this patient had total points of 192 according to our pneumonia risk prediction model, with a pneumonia risk prediction probability of >0.9. The patient underwent a chest CT, which showed multiple lamellar hyperintensities in multiple lobes of both lungs with solid lesion density, bronchial air sign within, and halo sign at the edges of some lesions.

We further explored the correlation between the predictive values of the pneumonia risk prediction model constructed in this study and the actual pneumonia severity rating. As mentioned in the method, patients with pneumonia were also classified into grades 0, 1, 2, 3, and 4 according to the extent and distribution of lung involvement (no lung involvement was categorized as grade 0). The actual pneumonia rating results are shown in Table 4. A positive linear correlation was found between the predicted pneumonia probability of the pneumonia risk model and actual pneumonia grade using GAM (Figure 5); see Figure 6 for examples.

## 4. Discussion

In this study, we constructed a pneumonia risk prediction model based on common, easily obtainable, and inexpensive clinical indicators such as “age,” “InCRP,” and “%Mon” to classify the pneumonia risk of patients infected with SARS-CoV-2. It provides an appropriate reference for clinicians in selecting chest CT examinations to reduce unnecessary medical ionizing radiation and alleviate patients' economic burden. The model performs well in discrimination, calibration, and clinical effectiveness and can be widely applied for clinical use.

### 4.1. Analysis of the Rationality of Including “Age” in the Pneumonia Risk Prediction Model in This Study

The severity and fatality rates of COVID-19 significantly vary with age group, and they rise sharply in older people [18–20]. According to recent studies, the activation of the nucleotide-binding domain and leucine rich repeat containing family, pyrin domain containing 3 (NLRP3) inflammasome, plays a role in lung inflammation and fibrosis induced by SARS-CoV-2 infections [21]; the NLRP3 inflammasome is excessively activated in older individuals due to impaired mitochondrial function, elevated levels of mitochondrial reactive oxygen species (mtROS), and/or mitochondrial DNA. This results in an exaggerated response from classically activated macrophages and subsequent increases in IL-1*β* [22]. This explains, to some extent, why elderly patients are more likely to have pneumonia after being infected with SARS-CoV-2 and also provides evidence for the rationality of including age as a predictive factor in our prediction model.

### 4.2. Analysis of the Rationality of Including “InCRP” in the Pneumonia Risk Prediction Model in This Study

As a general indicator of inflammation, CRP is associated with the clinical severity of COVID-19 [20, 23, 24]. CRP is an inflammatory biomarker synthesized by the liver. Our results show that CRP levels are significantly elevated in SARS-CoV-2-infected individuals, which is consistent with previous research [24, 25], and it may indicate COVID-19 changes earlier than chest CT—CRP was significantly elevated before CT findings in severe COVID-19 patients [26].

### 4.3. Analysis of the Rationality of Including “%Mon” in the Pneumonia Risk Prediction Model in This Study

In our study, %Mon was partially associated with the risk of pneumonia, which is in accord with recent studies [27]. Monocytes are innate immune system cells that participate in several immune function events, including phagocytosis, antigen presentation, and inflammatory responses [28]; circulating monocytes extravasate into peripheral tissues during sterile and nonsterile inflammation and undergo differentiation into macrophages or dendritic cells. A previous review article discussed the buildup of monocyte/macrophage cells in the lungs. These cells are likely sources of the proinflammatory cytokines and chemokines linked to deadly diseases brought on by human coronavirus infections, such as COVID-19 [29]. It suggests that the migration of monocytes into lung tissue may be the cause of the monocyte reduction in peripheral blood.

In previous relevant studies, additional factors, such as cardiovascular disease, hypertension, chronic respiratory disease, diabetes, obesity, and high serum ferritin levels, were found to be associated with the progression of COVID-19 [30–32]. Angiotensin-converting enzyme 2 (ACE2) has been found to be a pathway by which SARS-CoV-2 enters cells, and angiotensin-converting enzyme inhibitor (ACE1) and angiotensin II receptor antagonist (ARB) are mainly used to treat cardiovascular disease and hypertension, which may lead to increased ACE2 expression and promote SARS-CoV-2 infection in hypertensive patients [33]. Moreover, smokers and COPD patients have higher levels of ACE2 expression in their lungs [34, 35]. This may go some way towards explaining why patients with chronic respiratory disease are more likely to progress after SARS-CoV-2 infection. Diabetes patients are more likely to develop COVID-19 at a severe stage. This might be brought on by hyperglycemic circumstances that affect neutrophil activity, antioxidant system function, and humoral immunity, all contributing to immunological dysfunction [36]. Obesity affects lung function by influencing lung volume and compliance, as well as narrowing peripheral airways [37]. Additionally, due to the high expression of angiotensin-converting enzyme type 2 in adipose tissue compared to the lungs, there is a hypothesis that SARS-CoV-2 may be capable of entering adipocytes and causing infection. This could contribute to the spread of the virus to other organs or serve as a natural reservoir for prolonged viral clearance [38]. Clinically applicable inflammatory marker panels now contain ferritin. Inflammation can cause the release of ferritin from macrophages or cells owing to tissue damage. This release explains the abnormal levels of ferritin in inflammation. Since our study is based on a retrospective analysis, it is limited because of missing information, so some of the valuable indicators reported by relevant studies are not included in this study. In addition, some of the indicators were not included in our study because they were derived from patients' complaints rather than standard medical diagnoses and thus had low credibility.

From the standpoint of model promotion, the more streamlined a prediction model is, the less expensive, easier to use, and more suited to wide application it is. However, it will also result in a decline in prediction performance.

This is a matter of balance: whether the model should be applied mainly for primary screening of high-risk cases or whether it should prefer higher predictive accuracy. It depends on the application scenario of the constructed model.

In this study, the pneumonia risk prediction model we constructed was mainly applied to the primary screening of people at high risk of pneumonia in SARS-CoV-2-infected individuals, so we chose a more streamlined modeling strategy.

One unexpected finding was that the model performed better in the validation cohort than in the training cohort. This result may be explained by the relatively small sample size of the validation cohort and a certain degree of homology with the training cohort.

## 5. Conclusion

In this study, a pneumonia risk prediction model was developed and externally validated based on simple clinical and blood test indicators. The model was used to diagnostically predict the likelihood of pneumonia in patients infected with SARS-CoV-2 and performed well on dimensions of discrimination, calibration, and clinical validity. It can be used as a reference for the management of pneumonia risk classification in SARS-CoV-19-infected patients.

## 6. Limitations of This Study

Our study has several limitations. First, despite applying the inclusion criteria strictly, we could not completely rule out cases with potential lesions in body parts other than the lungs from influencing the predictors at study entry. This caused some confusion in constructing the model and difficulties in evaluating its predictive performance.

Second, even though external validation was carried out, it was a single-center retrospective study, and the sample size was somewhat tiny.

In later research, larger-sample and multicenter studies would be required to calibrate and validate the model.

## Figures and Tables

**Table 1:** Baseline indicators in the training cohort and validation cohort.

Characteristic	Training cohort			Validation cohort		
	No pneumonia	Incident pneumonia	*P* value	No pneumonia	Incident pneumonia	*P* value
Participants	99	106		47	47	
Age (year)	32.00 (14.00–86.00)	61.50 (17.00–97.00)	<0.001	39.00 (17.00–79.00)	69.00 (2.00–89.00)	<0.001
Gender			0.718			0.668
Female	52 (52.53%)	53 (50.00%)		29 (61.70%)	31 (65.96%)	
Male	47 (47.47%)	53 (50.00%)		18 (38.30%)	16 (34.04%)	
CRP (mg/L)	6.82 (0.00–154.30)	18.60 (0.47–359.14)	<0.001	1.66 (0.00–115.27)	28.21 (0.00–236.13)	<0.001
WBC (10^9^/L)	5.40 (1.41–10.36)	5.53 (2.36–15.56)	0.038	7.35 (2.43–9.42)	6.82 (2.54–14.64)	0.511
RBC (10^12^/L)	4.86 (3.31–6.44)	4.79 (2.78–6.55)	0.178	4.90 (3.84–6.29)	4.46 (3.30–5.62)	<0.001
HGB (g/L)	146.00 (67.00–184.00)	145.00 (93.00–192.00)	0.224	141.00 (116.00–173.00)	132.00 (100.00–171.00)	0.002
PLT (10^9^/L)	185.00 (98.00–361.00)	173.00 (51.00–460.00)	0.090	269.00 (136.00–424.00)	221.00 (59.00–579.00)	0.008
%Neu (%)	69.10 (29.00–89.30)	70.65 (38.80–94.10)	0.019	61.30 (44.70–82.10)	71.40 (44.70–92.60)	<0.001
%Lymph (%)	19.70 (4.40–60.60)	20.00 (2.40–53.80)	0.189	30.70 (8.90–47.60)	19.40 (5.80–47.00)	<0.001
%Mon (%)	9.40 (3.70–29.50)	7.90 (1.40–20.10)	<0.001	7.00 (3.40–14.50)	7.20 (1.50–19.30)	0.295
%Eos (%)	0.60 (0.00–8.00)	0.25 (0.00–5.90)	0.016	1.20 (0.00–11.20)	0.70 (0.00–3.20)	0.017
#Eos (10^9^/L)	0.03 (0.00–0.35)	0.01 (0.00–0.36)	0.057	0.08 (0.00–0.91)	0.04 (0.00–0.23)	0.022
%Bas (%)	0.20 (0.00–7.50)	0.10 (0.00–0.80)	0.002	0.20 (0.00–0.70)	0.10 (0.00–0.40)	0.004
#Bas (10^9^/L)	0.01 (0.00–0.32)	0.01 (0.00–0.03)	0.049	0.01 (0.00–0.04)	0.01 (0.00–0.02)	0.001
#Neu (10^9^/L)	3.45 (0.72–8.25)	3.75 (0.91–14.36)	0.012	4.26 (1.47–6.85)	4.67 (1.46–12.80)	0.188
#Lymph (10^9^/L)	1.05 (0.23–3.56)	1.06 (0.24–5.47)	0.864	2.05 (0.73–3.34)	1.19 (0.55–3.94)	<0.001
#Mon (10^9^/L)	0.48 (0.19–1.50)	0.43 (0.08–1.51)	0.216	0.48 (0.17–0.74)	0.52 (0.14–1.14)	0.388
HCT (%)	42.10 (22.40–52.10)	41.15 (26.00–53.30)	0.135	42.50 (36.20–52.00)	39.80 (30.90–49.00)	<0.001
MCV (fL)	87.60 (55.30–98.10)	87.55 (62.00–99.40)	0.597	89.30 (66.10–105.00)	89.70 (69.30–102.50)	0.427
MCHC (g/L)	350.00 (301.00–369.00)	352.00 (298.00–376.00)	0.147	331.00 (311.00–352.00)	333.00 (315.00–353.00)	0.198
MCH (Pg)	30.70 (16.70–33.90)	30.90 (20.10–35.20)	0.286	30.10 (21.00–34.40)	30.10 (22.30–35.30)	0.276
RDW-SD (fL)	39.40 (33.30–55.50)	40.15 (33.70–49.60)	0.040	40.50 (36.00–48.50)	40.20 (32.50–49.90)	0.771
RDW-CV (%)	12.20 (11.30–19.90)	12.40 (11.30–17.40)	0.096	12.10 (11.10–15.90)	12.20 (10.90–14.00)	0.803
PDW (%)	16.20 (10.20–17.10)	16.30 (15.40–17.80)	0.014	16.20 (15.60–16.80)	16.30 (15.70–17.40)	0.112
MPV (fL)	9.50 (7.30–11.90)	9.80 (8.00–12.40)	0.020	9.30 (7.70–11.70)	9.50 (7.20–12.30)	0.122
PCT (%)	0.18 (0.10–0.34)	0.17 (0.05–0.41)	0.247	0.25 (0.14–0.36)	0.20 (0.06–0.53)	0.010
P-LCR (%)	23.80 (8.80–41.90)	26.10 (12.40–44.90)	0.033	21.70 (12.60–39.20)	24.20 (10.00–42.60)	0.226

CRP = C reactive protein; WBC = white blood cells; RBC = red blood cells; HGB = hemoglobin; PLT = platelets; %Neu = neutrophils (percentage); %Lymph = lymphocytes (percentage); %Mon = monocytes (percentage); %Eos = eosinophils (percentage); #Eos = eosinophils (number); %Bas = basophils (percentage); #Bas = basophils (number); #Neu = neutrophils (number); #Lymph = lymphocytes (number); #Mon = monocytes (number); HCT = hematocrit; MCV = mean corpuscular volume; MCHC = mean corpuscular hemoglobin concentration; MCH = mean corpuscular hemoglobin; RDW-SD = red cell distribution width-standard deviation; RDW-CV = red cell distribution width-coefficient of variation; PDW = platelet distribution width; MPV = mean platelet volume; PCT = plateletcrit; P-LCR = platelet large cell ratio.

**Table 2:** Univariate and multivariate logistic regression analyses of candidate predictors of pneumonia risk prediction models in the training cohort.

Characteristic	Univariate analysis		Multivariate analysis	
	*β* (95% CI)/OR (95% CI)	*P* value	*β* (95% CI)/OR (95% CI)	*P* value
Gender		0.7178		
Female	1.0			
Male	1.11 (0.64, 1.91)			
Age (year)	1.03 (1.02, 1.04)	<0.0001	**1.03 (1.01, 1.05)**	**0.0095**
WBC (10^9^/L)	1.18 (1.04, 1.35)	0.0136	1.38 (0.46, 4.09)	0.5663
RBC (10^12^/L)	0.70 (0.45, 1.07)	0.0978	2.77 (0.68, 11.32)	0.1549
HGB (g/L)	0.99 (0.98, 1.01)	0.1937		
PLT (10^9^/L)	1.00 (0.99, 1.00)	0.1753		
%Neu (%)	1.03 (1.01, 1.05)	0.0051	**0.76 (0.59, 0.99)**	**0.0415**
%Lymph (%)	0.98 (0.96, 1.00)	0.0769	0.80 (0.61, 1.04)	0.0991
%Mon (%)	0.85 (0.77, 0.93)	0.0005	**0.66 (0.50, 0.88)**	**0.0041**
#Neu (10^9^/L)	1.23 (1.06, 1.42)	0.0056	0.98 (0.24, 3.96)	0.9818
#Lymph (10^9^/L)	0.99 (0.65, 1.51)	0.9585		
#Mon (10^9^/L)	0.53 (0.17, 1.65)	0.2730		
HCT (%)	0.95 (0.90, 1.01)	0.0989	0.88 (0.75, 1.04)	0.1332
MCV (fL)	1.01 (0.97, 1.05)	0.5854		
MCHC (g/L)	1.02 (0.99, 1.04)	0.2370		
MCH (Pg)	1.05 (0.94, 1.16)	0.3793		
RDW-SD (fL)	1.09 (1.00, 1.19)	0.0522	0.95 (0.81, 1.12)	0.5666
RDW-CV (%)	1.18 (0.90, 1.56)	0.2336		
PDW (%)	2.35 (1.15, 4.82)	0.0195	1.44 (0.65, 3.16)	0.3659
MPV (fL)	1.53 (1.11, 2.11)	0.0097	18.62 (0.98, 355.15)	0.0519
PCT (%)	0.14 (0.00, 22.97)	0.4483		
P-LCR (%)	1.05 (1.01, 1.10)	0.0178	0.70 (0.47, 1.04)	0.0773
InCRP (mg/L)	1.84 (1.46, 2.32)	<0.0001	**1.89 (1.40, 2.55)**	**<0.0001**
%Eos (%)	0.83 (0.68, 1.01)	0.0594	0.71 (0.50, 1.03)	0.0691
#Eos (10^9^/L)	0.09 (0.00, 3.02)	0.1814		
%Bas (%)	0.08 (0.01, 0.49)	0.0058	2.95 (0.97, 90.23)	0.5346
#Bas (10^9^/L)	0.00 (0.00, 0.01)	0.0249	0.00 (0.00, inf.)	0.3203

WBC = white blood cells; RBC = red blood cells; HGB = hemoglobin; PLT = platelets; %Neu = neutrophils (percentage); %Lymph = lymphocytes (percentage); %Mon = monocytes (percentage); #Neu = neutrophils (number); #Lymph = lymphocytes (number); #Mon = monocytes (number); HCT = hematocrit; MCV = mean corpuscular volume; MCHC = mean corpuscular hemoglobin concentration; MCH = mean corpuscular hemoglobin; RDW-SD = red cell distribution width-standard deviation; RDW-CV = red cell distribution width-coefficient of variation; PDW = platelet distribution width; MPV = mean platelet volume; PCT = plateletcrit; P-LCR = platelet large cell ratio; InCRP = natural log-transformed value of CRP; %Eos = eosinophils (percentage); #Eos = eosinophils (number); %Bas = basophils (percentage); #Bas = basophils (number). Significance in bold in Table 2 indicates indicators with p values less than 0.05 in multivariate analysis.

**Table 3:** Diagnostic parameters of the pneumonia risk model.

Variable	Value	
	Training cohort	Validation cohort
AUC	0.7820 (95% CI: 0.7254, 0.8439)	0.8432 (95% CI: 0.7588, 0.9151)
Cutoff value	0.5	0.5
Specificity	66.33%	73.91%
Sensitivity	70.75%	76.09%
Accuracy	68.63%	75.00%
Positive-LR	2.10	2.92
Negative-LR	0.44	0.32
Diagnose-OR	4.77	9.02
Positive-pv	69.44%	74.47%
Negative-pv	67.71%	75.56%

**Table 4:** Distribution of actual pneumonia grades in the training cohort and validation cohort.

Actual pneumonia grade	Participants *n* (%)	
	Training cohort	Validation cohort
0	99 (48.29%)	47 (50.00%)
1	70 (34.15%)	22 (23.40%)
2	23 (11.22%)	18 (19.15%)
3	12 (5.85%)	6 (6.38%)
4	1 (0.49%)	1 (1.06%)

## Data Availability

The data used to support the findings of this study are restricted by the Medical Ethics Committee of Hunan Provincial People's Hospital (The First Affiliated Hospital of Hunan Normal University) to protect the patient. Data are available from Xi Yi, beimingyi0322@163.com, for researchers who meet the criteria for access to confidential data.
